# Impact papers on aging in 2009

**DOI:** 10.18632/aging.100132

**Published:** 2010-03-23

**Authors:** Mikhail V. Blagosklonny, Judy Campisi, David A. Sinclair, Andrzej Bartke, Maria A. Blasco, William M. Bonner, Vilhelm A. Bohr, Robert M. Brosh Jr, Anne Brunet, Ronald A. DePinho, Lawrence A. Donehower, Caleb E. Finch, Toren Finkel, Myriam Gorospe, Andrei V. Gudkov, Michael N. Hall, Siegfried Hekimi, Stephen L. Helfand, Jan Karlseder, Cynthia Kenyon, Guido Kroemer, Valter Longo, Andre Nussenzweig, Heinz D. Osiewacz, Daniel S. Peeper, Thomas A. Rando, K Lenhard Rudolph, Paolo Sassone-Corsi, Manuel Serrano, Norman E. Sharpless, Vladimir P. Skulachev, Jonathan L. Tilly, John Tower, Eric Verdin, Jan Vijg

**Affiliations:** ^1^ Roswell Park Cancer Institute, Buffalo, NY, USA; ^2^ Buck Institute for Age Research, Novato, CA, USA; ^3^ Harvard Medical School, Boston, MA, USA; ^4^ Southern Illinois University, Springfield, IL, USA; ^5^ Spanish National Cancer Center, Madrid, Spain; ^6^ National Cancer Institute, NIH, Bethesda, MD, USA; ^7^ National Institute on Aging, NIH, Baltimore, MD, USA; ^8^ Stanford University, Stanford, CA, USA; ^9^ Dana-Farber Cancer Institute, Boston, MA, USA; ^10^ Baylor College of Medicine, Houston, TX, USA; ^11^ University of Southern California, Los Angeles, CA USA; ^12^ NHLBI, NIH, Bethesda, MD, USA; ^13^ University of Basel, Basel, Switzerland; ^14^ McGill University, Montreal, Canada; ^15^ Brown University, Providence, RI, USA; ^16^ The Salk Institute, La Jolla, CA, USA; ^17^ University of California, San Francisco, CA, USA; ^18^ INSERM, U848, Villejuif, France; ^19^ Goethe University Frankfurt, Frankfurt, Germany; ^20^ The Netherlands Cancer Institute, Amsterdam, Netherlands; ^21^ Medical School Hannover, Hannover, Germany; ^22^ University of California, Irvine, CA, USA; ^23^ University of North Carolina, Chapel Hill, NC, USA; ^24^ Moscow State University, Moscow, Russia; ^25^ Massachusetts General Hospital, Boston, MA, USA; ^26^ Albert Einstein College of Medicine, Bronx, USA

**Keywords:** aging, senescence, signal transduction, genes for longevity

## Abstract

The editorial board of *Aging* reviews research papers published in 2009, which they believe have or will have a significant impact on aging research.  Among many others, the topics include genes that accelerate aging or in contrast promote longevity in model organisms, DNA damage responses and telomeres, molecular mechanisms of life span extension by calorie restriction and pharmacologic interventions into aging.  The emerging message in 2009 is that aging is not random but determined by a genetically-regulated longevity network and can be decelerated both genetically and pharmacologically.

## Telomeres

The 2009 Nobel Prize in Physiology or Medicine was awarded to Elizabeth Blackburn, Carol Greider and Jack Szostak for their contributions to our understanding of how the ends of eukaryotic chromosomes, telomeres, are maintained by a specialized reverse transcriptase, telomerase.  This award is the closest Nobel Prize to date related to aging. Of course, the major significance of the work relates to basic cell biology and cancer, rather than aging research.  In fact, whereas telomere shortening explains the Hayflick limit (replicative senescence) in human cells, it cannot explain the difference in longevity between mice and men.  But there may be other links between telomeres and aging.  In 2009, several publications by Epel, Blackburn and co-workers provide a new link between telomere length and age-related diseases.  As published in the first issue of *Aging*, the rate of telomere shortening in peripheral leukocytes predicts mortality from cardiovascular disease in elderly men [1].  Even more intriguingly, pessimism correlates with short leukocyte telomeres and elevated interleukin (IL)-6 in post-menopausal women [2].  The cause-and-effect relationship between telomere length and these physiological endpoints is unknown, but several non-mutually exclusive explanations can be proposed.  Rapid telomere shortening may indicate a cellular hyper-activation, hyper-proliferation and/or hyper-secretory phenotypes often associated with cellular senescence, stem cell exhaustion and diseases of aging.

In agreement with these possibilities, telomere length was shown to regulate the expression of interferon-stimulated gene 15 (ISG15). Short-telomeres up-regulated ISG15 independent of DNA damage signaling.  This finding demonstrated for the first time that an endogenous human gene can be regulated by telomere length prior to the onset of telomere dysfunction and DNA damage signals.  It was suggested that the upregulation of ISG15 by telomere shortening may contribute to the chronic inflammation associated with human aging [3].  Pertinent to this idea, also in 2009, the secretion of inflammatory cytokines such as IL-6 and IL-8 by senescent cells, whether made senescent by dysfunctional telomeres or DNA damage, was shown to be suppressed by two micro-RNAs (miR-146a and 146b) [4]. It was proposed that these micro-RNAs modulate inflammatory responses by affecting signal transduction pathways that contribute to a larger senescence associated secretory phenotype.  It will be of interest to know whether miR-146a/b also suppresses ISG15 expression, and if this effect is influenced by telomere status.

It was also demonstrated that dysfunction of a telomere-binding protein is sufficient to produce severe telomeric damage in the absence of telomere shortening, resulting in premature tissue degeneration and development of neoplastic lesions [5]. New insight has been gained in the understanding of how telomeres are maintained and how the processes of DNA repair occur in telomeres.  For example, it appears that the guardians of the genome, the RecQ helicases, actively participate in this repair process [6].

Damaged telomeres were also found to be the major factor contributing to the wide variability in the amount of DNA damage signaling in human tumor cell lines, findings that may help clarify the relative contributions of non-telomeric DNA double-strand breaks and damaged telomeres to levels of genomic instability [7].

## DNA damage response and aging

In 2009, it was demonstrated that the persistent (but not transient) DNA damage response (DDR) associated with senescent cells is essential for their ability to express and secrete inflammatory cytokines [8]. Cell surface-bound IL-1alpha is essential for the senescence-associated secretion of IL-6 and IL-8, 2 proinflam-matory cytokines, reinforcing the senescence phenotype [9].

Both the initiation and maintenance of cytokine secretion required the DDR proteins ATM, NBS1 and CHK2, but not p53.  ATM was also essential for IL-6 secretion during oncogene-induced senescence and by damaged cells that bypass senescence.  It was proposed that this activity of the DDR allows senescent cells to communicate their compromised state to the surrounding tissue [8].  In addition, a DDR may occur in senescent cells even in the absence of detectable DNA damage [10].  This pseudo-DDR is a marker of cellular hyperactivation and is inhibited by rapamycin [10], a clinically approved drug that decelerates cellular senescence [11]. Thus, persistent DDR signaling, regardless of DNA damage, may be a part of the senescent phenotype.

It was shown that longevity extension mutations in the yeast SCH9, the yeast homolog of the conserved pro-aging gene S6K (Ribosomal Protein S6 Kinase), caused a major reduction in age-dependent DNA damage by lowering the activity of error-prone DNA repair genes [12].

Also, age-dependent deterioration of nuclear pore complexes causes an increase in nuclear permeability and the leaking of cytoplasmic proteins into the nucleus in postmitotic cells [13].

The ability to respond to stress decreases with age.  Stress-responding factors which regulate transcription can influence longetivity.  In 2009, Westerheide et al demonstrated that stress-induced regulation of heat shock factor 1 (HSF-1) by the deacetylase SIRT1 (sirtuin 1) may play a role in the regulation of life span [14].  Defining the targets of sirtuins may help to understand the importance of transcriptional regulation in age-related diseases.

An intriguing possibility is that the response of the cells to certain types of DNA damage (e.g. DNA breaks) results in epigenetic changes that alter gene expression [15].  These changes do occur in mammals and it will be interesting to test whether these epigenetic changes in response to DNA damage are associated with, or can actually cause aging.

## Mitochondria, oxidative stress and aging

 On the other hand, the free radical theory, which posits that aging is caused by an *accumulation* of oxidative damage, was critically questioned in 2009. First, overexpression of major antioxidant enzymes, which decrease free radicals, did not extend the lifespan of mice [16].  Second, deletion of mitochondrial superoxide dismutase (Sod-2) extended life span in *Caenorhabditis elegans* [17].  Third, life span extension by dietary restriction was not linked to protection against somatic DNA damage in *Drosophila melanogaster* [18].  Fourth, Sod-2 haploinsufficiency did not accelerate murine aging, even in mice with dysfunctional telomeres [19]. In addition it was demonstrated that the reduced energy metabolism and the increased oxidative stress in the mitochondria of young Mclk1+/- mice results in an almost complete protection from the age-dependent loss of mitochondrial function. Moreover, this altered mitochondrial condition is linked to a significant attenuation of the rate of development of oxidative biomarkers of aging. Thus, this study indicates that mitochondrial oxidative stress is not causal to aging [20]. It was reported that RNAi of five genes encoding components of mitochondrial respiratory complexes I, III, IV, and V leads to increased life span in flies. Long-lived flies with reduced expression of electron transport chain (ETC) genes do not consistently show reduced assembly of respiratory complexes or reduced ATP levels. In addition, extended longevity is not consistently correlated with increased resistance to the free-radical generator paraquat [21].

These results are in agreement with previous papers showing that antioxidants overexpression causes minor effects in life span extension in yeast, flies, and mice compared to those caused by mutations in signal transduction genes. It is likely that increase protection against superoxide must be accompanied by a number of other changes to be effective in life span extension. For instance, LON, a AAA protease located in the mitochondrial matrix, increases stress tolerance, mitochondrial oxygen consumption, while decreasing oxidative damage of proteins in the fungal aging model *Podospora anserine* [22]. In the same model organism, deletion of a gene encoding a O-methyltransferase, which decrease levels of reactive oxygen species, leads to a decreased lifespan [23].

## Calorie restriction (CR)

Caloric restriction (CR) without malnutrition delays aging and extends life span in diverse species; however, its effect in primates had not been clearly established.  In 2009, a 20-year longitudinal study of adult-onset CR in rhesus monkeys demonstrated that moderate CR lowered the incidence of aging-related deaths.  At the time point reported, 50% of control animals had survived, compared with 80% of CR animals.  CR delayed the onset of several age-associated pathologies such as diabetes, cancer, cardiovascular disease and brain atrophy [24]. The CR trial in primates raised hope that CR might be effective in humans.

In 2009, numerous studies continued to establish links between caloric restriction (CR) and longevity signaling pathways, including Sir2 (sirtuin) and p53 in *D. melanogaster* [25] and the E3 ubiquitin ligase WWP-1 in *C. elegans* [26] as well as upstream and downstream components of the TOR (Target of Rapamycin) pathway: RHEB-1 in *C. elegans* [27], Tor1 and Sch9 (a homolog of the mammalian kinases Akt and S6K) in yeast [28], and 4E-BP (Eukaryotic Translation Initiation Factor 4E Binding Protein) in *Drosophila* [29]. It was shown that glucose shortens the life span of C. elegans by downregulating DAF-16/FOXO activity and aquaporin gene expression [30]. In addition, the HIF (hypoxia inducible factor) pathway was implicated in aging and longevity in *C. elegans* [31,32]. The different results of two studies have been in general reconciled [33]. In 2009, it has also been shown that in C. elegans CR is mediated by a network of independent, but overlapping pathways [34], suggesting a ‘CR network'. Notably, neuronal SIRT1 regulated endocrine and behavioral responses to CR [35].

It has been shown that disruption of growth hormone receptor (GHR) prevents calorie restriction from improving insulin action and longevity [36]. In normal mice, CR increased insulin sensitivity in liver and muscle. In GHRKO mice, intrinsic insulin-sensitivity could be attributed to a reduction of inhibitory serine phosphorylation of IRS-1 (Insulin receptor substrate 1) in muscle. CR failed to further increase insulin signaling (insulin sensitivity) in GHRKO mice as compared to normal mice, likely explaining the absence of CR effects on longevity in these long-lived mice [36].

Finally, it was tested whether reallocation of nutrients from reproduction to somatic maintenance could explain the life extending effect of CR. If this were the case, long life under dietary restriction and high fecundity (reproduction) under full feeding would be mutually exclusive. Adding methionine alone to the dietary restriction condition was necessary and sufficient to increase fecundity as much as did full feeding, but without reducing lifespan. Reallocation of nutrients therefore does not explain the responses to dietary restriction. In contrast, reduced activity of the insulin/insulin-like growth factor signaling protected against the shortening of lifespan with full feeding [37].

## Pharmacologic intervention

The ultimate goal of biomedical research is the development of therapeutic drugs. As shown previously, activation of mTOR (mammalian Target of Rapamycin) is required for acquiring senescent phenotype in p21-arrested human cells, whereas deactivation of mTOR converts senescence into quiescence. In 2009, it was further demonstrated that the inhibitor of mTOR rapamycin decelerated cellular senescence of p21-arrested human and mouse cells [11]. Similarly, inhibitors of PI-3K and MEK, LY-294002 and U0126, deactivated mTOR and suppressed cellular senescence (converting it into quiescence) [38], defining direct and indirect mTOR inhibitors as aging-suppressants or gero-suppressants.

The most striking event of the year was the demonstration that rapamycin, administrated to middle-aged (600 day old) mice, significantly extended their life span [39]. The effect was seen at three independent test sites in genetically heterogeneous mice, chosen to avoid genotype-specific effects on disease susceptibility [39]. Rapamycin also prolonged the life of 22-month old mice [40]. [Note: publications by Bjedov et al (Cell Metab 2010 Jan) and by Moskalev and Shaposhnikov (coming in print 2010) that rapamycin extends life span in *Drosophila* will be reviewed next year].

It was shown that clioquinol, a metal chelator that has beneficial effects in several models of neuro-degenerative diseases, inhibits the activity of the mitochondrial enzyme CLK-1 in mammalian cells. Clioquinol-treated nematodes and mice presented a variety of phenotypes produced by mutational reduction of CLK-1. Given that reduction of CLK-1 slows down aging in these organisms, these results suggest that clioquinol (by inhibiting CLK-1) may slow down the aging process [41].

Finally, as a follow-up of the work on the anti-aging effects of mitochondria-targeted antioxidant SkQ1 [42], it was demonstrated that Sk inhibits age-dependent involution of the thymus in normal and senescence-prone rats [43].

## Stem cells and aging

In 2009, several lines of evidence suggested that overactivation of signaling pathways might cause exhaustion of stem cells and that vice versa ‘longevity genes' could prevent stem cell exhaustion.  Thus, mTOR mediated Wnt-induced epidermal stem cell exhaustion and aging phenotypes in skin [44].  Further, hyper-activation of mTORC1 caused hyper-proliferation and subsequent exhaustion of hematopoietic stem cells.  Pharmacological approaches showed that PTEN, TSC1 and PML regulate hematopoietic stem cell (HSC) maintenance through mTORC1 [45].  In addition, FOXO transcription factors were found to be necessary for adult neural stem cell homeostasis  [46,47]. Importantly, stem cell aging could be suppressed pharmacologically [40,44]. The PI3K-AKT-FoxO pathway is integral to lifespan regulation in lower organisms  plays a prominent role in neural stem/progenitor cell (NSC) proliferation and renewal. FoxO-deficient mice show initial increased brain size and proliferation of neural progenitor cells during early postnatal life, followed by precocious significant decline in the NSC pool and accompanying neurogenesis in adult brains [46].

In addition, functions of aging organs can be rejuvenated by young supporting stem cells.  As published in the first issue of *Aging*, once-monthly infusions of bone marrow (BM)-derived cells from young adult female mice sustained the fertility of aging females long past their time of normal reproductive failure [48].  The fertility-promoting effects were observed regardless of whether the infusions were initiated in young adult or middle-aged females, and were specific for bone marrow harvested from female donors.  This "rejuvenation" did not depend on the development of mature eggs from germline cells in the donor marrow, but from host germline cells sustained by the infusions [48,49]. In fact, very recent studies showed that aged mouse ovaries lacking oocytes retain a rare population of germline stem cells that, when transplanted into a young host ovarian environment, are able to generate immature oocytes contained within follicles [49]. Thus, reproductive failure with age may be due, at least in part, to deterioration of somatic microenvironments (niches) that support stem cell function.

## Nuclear reprogramming and senescence

Much interest has also been devoted in the past year to nuclear reprogramming of differentiated cells into induced pluripotent stem (iPS) cells by using defined factors. Understanding which factors facilitate the reprogramming process is thought to give clues to the process of carcinogenesis. Inversely, nuclear reprogramming could be also envisioned as a "rejuvenation process". In this regard, p53 and p16^INK4a^ tumor suppressor proteins were shown to be important in limiting reprogramming [50-55].  Activation of p53 was suggested to be more important in murine cells, whereas activation of p16^INK4a^ appeared the predominant barrier in human cells [50].

Of particular importance to the field of regenerative medicine, which will need patient-specific stem cells derived from older patients, is reprogramming efficiency in fibroblasts from aged humans versus young humans.  There is an age-associated decline in reprogramming efficiency, which is largely reversed by inactivation of the p16^INK4a^ tumors suppressor gene, whose expression is increased markedly with aging in several human and murine tissues [50,55].  Along these lines, it was shown that the increased expression of p16INK4a with aging could be measured on human peripheral blood samples, and that an individual's p16INK4a expression was a good biomarker of their "molecular age" [56]. The same group also provided further understanding of the observed linkage of SNPs near the *CDKN2a/b* locus (which encodes the p16^INK4a^, p15^INK4b^ and ARF tumor suppressors) with human atherosclerotic disease [57].  Expression of *CDKN2a/b* transcripts is decreased in individuals harboring the risk alleles, suggesting that atherosclerotic disease may result from aberrant, unrestrained proliferation. In this regard, studies on mice overexposing the CDKN2a/b locus were found to have delayed aging and extended longevity [58].

## Genetics of aging

In 2009, numerous publications extended our knowledge on the role of sirtuins [35], TOR signaling [59,60], and the stress response factors HSF-1 and DAF-16 [61] in aging.  Of particular importance, it was shown that deletion of the gene encoding Ribosomal Protein S6 Kinase 1 (S6K1) and disruption of PKA extend the life span of mice [62,63], whereas the gene encoding Eukaryotic Translation Initiation Factor 4E Binding Protein (4E-BP) was shown to be essential for life span extension by CR in *Drosophila* [29].  Moreover, 4E-BP was shown to act downstream of TOR to modulate cardiac aging in *Drosophila* [64].  Finally, SIRT6 was shown to play a critical role in DNA double-strand break repair [65].

In 2009, Kenyon and co-workers further uncovered mechanisms of their previous observations made in 1999 (Hsin and Kenyon, Nature, 1999, 399:362-6) that in *C elegans* and *Drosophila* the aging of the soma is influenced by the germline: namely, when germline-stem cells are removed, aging slows and lifespan is increased. In 2009, it was published that a predicted transcription elongation factor, TCER-1, plays a key role in this process [66]. When the germ cells are removed, the levels of TCER-1 rise in somatic tissues. This increase is sufficient to trigger key downstream events, as overexpression of tcer-1 extends the lifespan of normal animals that have an intact reproductive system. Intriguingly, TCER-1 specifically links the activity of a broadly deployed transcription factor, DAF-16/FOXO, to longevity signals from reproductive tissues [66]. In mice, Foxo1 integrates insulin signaling with mitochondrial function, and inhibition of Foxo1 can improve hepatic metabolism during insulin resistance and the metabolic syndrome [67].

A prior  work by Willcox et al (PNAS 2008, 105: 13987) showed that genetic variation within the FOXO3A gene was strongly associated with human longevity. Long-lived men also presented several additional phenotypes linked to healthy aging, including lower prevalence of cancer and cardiovascular disease, and high physical and cognitive function. Long-lived men also exhibited greater insulin sensitivity associated with homozygosity for the FOXO3A GG genotype. In 2009, confirming the Willcox observation, the flurry of papers showed the association between SNPs in the FoxO3A gene and extreme longevity in Japanese, German, American, Italian, and Chinese populations [68-71].

 There were intriguing publications on the complex role of p53 in longevity.  In *Drosophila melanogaster*, p53 exerted developmental stage-specific and sex-specific effects on adult life span, indicative of sexual antagonistic pleiotropy [72,73].  Further, an association between single nucleotide polymorphisms (SNPs) in p53 pathway genes and human fertility suggested that p53 regulates the efficiency of human reproduction.  These results provide a plausible explanation for selective pressure to retain some alleles in the p53 pathway, and suggest that such alleles are a good example of antagonistic pleiotropy [74].

Interestingly, SNPs in the p21 gene correlated with longevity in an Italian population [75]. Several papers have highlighted an important role of p53 in tissue fitness through its impact in preventing mobilization of stem cells harboring persistent DNA damage (ie, dysfunctional telomeres) [76,77]. However, the phenotypic outcome was tissue and context specific. In mouse epidermis deletion of p53 rescued organ maintenance and body fitness of neborn mice with dysfunctional telomeres [76]. In contrast, p53 deletion in the intestinal epithelium accelerated tissue dystruction and shortened the lifespan of aging telomere dysfunctional mice [77]. The latter phenotype was associated with aberrant survival chromosomal instable stem cell clones leading to abnormal differentiation and p53-independent apoptosis. The limitation of the survival of chromosomal instable stem cells is likely to represent a key step in the known role of p53 as a tumor suppressor. Also it was shown that the p53 family member, TAp63, is essential for maintenance of epidermal and dermal precursors and that, in its absence, these precursors senesce and skin ages prematurely [78].

Model systems continue to be instrumental in understanding the genetics of longevity.  The *WRN* gene defective in the premature aging disorder Werner syndrome encodes a protein with both helicase and exonuclease activities [79].  To dissect its genetic functions, human WRN was tested for its ability to rescue *sgs1*-related phenotypes.  WRN was shown to genetically interact with topoisomerase 3 and restore the slow growth phenotype of *sgs1 top3*.  WRN helicase but not exonuclease activity was genetically required for restoration of *top3* growth phenotype, demonstrating separation of function of WRN catalytic activities.  In a *top3* mutant background, DNA unwinding by WRN helicase may be deleterious to cell growth and genome homeostasis [80].

In 2009, a few studies delved into the genetics of the insulin-producing pancreatic beta-cell aging in humans and mice [81-83].  A loss of beta-cell replication with aging is a contributor to age-related increase in the incidence of type II diabetes.  Prior work had shown that p16^INK4a^ tumor suppressor causes an age-dependent decline in beta-cell replication.  In 2009, it was reported that loss of Polycomb (PcG) repression of p16INK4a mediated by the EZH2 histone methytransferase occurred with aging in humans and mice [82].  In mice, somatic deletion of EZH2 led to loss of beta-cell replication and diabetes, and these effects could be rescued by concomitant deletion of  p16^INK4a^ and Arf.

This work linked alterations of chromatin architecture with aging to expression of anti-proliferative molecules.  Bhushan and colleagues also reported a similar regulation of p16^INK4a^ expression with aging by the Bmi-1 PcG protein, which functions in concert with EZH2 to repress p16^INK4a^ expression [81]. Lastly, it was shown that p38MAPK activates p16I^NK4a^ with aging in beta-cells, suggesting a possible pharmacologic approach to regulating aging of this tissue [83].

## Autophagy

In 2009, the simple dogma that autophagy is always associated with or causes senescence was challenged. Although autophagy remains a crucial anti-aging mechanism, the relationship is likely to be complex.  Thus, autophagy was shown to be activated during cellular senescence, and activation correlated with negative feedback in the PI3K-mTOR pathway. A subset of autophagy-related genes was up-regulated during senescence: overexpression of one gene, ULK3, induced autophagy and senescence.  Furthermore, inhibition of autophagy delayed the senescence phenotype, including senescence-associated secretion.  These data suggest that autophagy, and its consequent protein turnover, may mediate acquisition of the senescence phenotype [84]. Inhibition of autophagy in adult *Drosophila* [85] or *C. elegans* [86] was found not to affect longevity, however autophagy was required for the increased life span caused by several pharmocologic and genetic manipulations in yeast, Drosophila and C. elegans [87-90], suggesting that autophagy may be limiting for life span under some conditions but not others.  Interestingly, resveratrol-mediated inhibition of mammalian S6 kinase by resveratrol suppressed autophagy [91].  In 2009, several reports further demonstrated that the TOR signaling pathway targets the Atg1/Atg13 protein kinase complex to control autophagy [92-94]. Furthermore, TOR-mediated autophagy regulates cell death in Drosophila neurodegenerative disease [95].

The natural polyanion spermidine can extend the chronological and replicative life span in yeast and increase the median and maximal longevity of fruit flies and nematodes (*C. elegans*). Spermidine was found to act as a potent inducer of autophagy in all species tested, including yeast, *Drosophila, C. elegans* [96].  The antiaging effect of spermidine was abolished by the deletion or depletion of essential autophagy genes in yeast, *Drosophila* and *C. elegans* [96]. In mice, a dietary supplementation with polyanions (including spermidine) also increases healthspan and lifespan [97], although the dependency of this phenomenon on autophagy has not been addressed yet. Spermidine likewise induces autophagy and longevity through its capacity to inhibit histone acetylases in yeast cells [96].

Sirtuin-1 and that of its *C. elegans* orthologue induce autophagy in human and nematode cells. Sirtuin-1 is also required for the induction of autophagy by its allosteric activator resveratrol (both in human cells and nematodes), culture in nutrient-free media (in human cells) and caloric restriction (in nematodes). In *C. elegans*, it was found that activation of Sirtuin-1 extended longevity in an autophagy-dependent fashion. Thus, the knockdown of the essential autophagy gene Beclin1/ATG6 abolished life span extension by Sirtuin-1 activation [87]. These results underscore the contribution autophagy to the regulation of longevity by pharmacological agents [98].

## Post-transcriptional gene regulation and aging

In fact, 2009 saw an escalation in interest in microRNAs and other non-coding RNAs implicated in aging and replicative senescence.  A prominent example of this regulation came studies of the mitogen-activated protein kinase (MAPK) signaling component MKK4 (MAPK kinase kinase 4).  MKK4 levels were elevated in aging tissues and in senescent cells thanks to reductions in the abundance of four microRNAs (miR-15b, miR-24, miR-25, and miR-141) that interacted with the 5'- and 3'-untranslated regions of the MKK4 mRNA and repressed its translation [99].

The other major class of post-transcriptional regulatory factors, RNA-binding proteins (RBPs), were also the focus of important age-related studies in 2009. Several RBPs that affect the turnover and translation of proteins implicated in proliferation, survival, inflammation, neurodegeneration, and cancer (HuR, AUF1, TIA-1, TTP) displayed elevated abundance in a broad array of human tissues and in all ages, suggesting that their influence extends throughout the human life span [100].  The RBP TTP (tristetraprolin) attracted especial attention because it triggered replicative senescence [101]; in keeping with the tumor-suppressive influence of replicative senescence, TTP was found to be eliminated in certain cancers [102].

## Circadian clock

There is growing evidence for a link between circadian rhythm,  signal-transduction genes, metabolism, cancer and aging [103,104].  The circadian clock gene *period* extended the health span of aging in *Drosophila melanogaster* [105].  Further, circadian control of the NAD+ salvage pathway by CLOCK-SIRT1 was demonstrated [106]. Intriguingly, light was found to activate MAPK (mitogen activated pathway kinase) in zebrafish cells, and this light-dependent activation controlled DNA repair [107]. In rats, circadian disruption induced by light-at-night accelerates aging and promotes tumorigenesis in rats [108]. In mice, it was reported that N-acetyl-L-cysteine (NAC), an antioxidant, ameliorated symptoms of premature aging associated with the deficiency of the circadian protein BMAL1 [109].

## Cancer and aging

CR is known to slow aging and delay cancer.  In 2009, it was reported that fasting abrogates side effects caused by chemotherapy in cancer patients.  Importantly, for those patients in whom cancer progression could be assessed, fasting did not prevent chemotherapy-induced reduction of tumor volume or tumor markers [110].  The link between aging and cancer via p53 was shown to be complex in 2009.  Thus, the ability of p53 to act as a defense against tumor progression was shown to be age-dependent [111].  Further, Levine and co-workers previously showed that p53 activity declines with age, and a recent study showed that p53 transcriptional activity is reduced in senescent cells [112]. Interestingly, SIRT1 knockout mice, which do not live longer when calorically restricted, were found to have normal rates of skin cancer but the ability of resveratrol, a SIRT1 activator, to protect the mice was greatly reduced [113], indicating that the anti-tumor activity of resveratrol is mediated at least in part by SIRT1.

Reduced incidence and delayed occurrence of fatal neoplastic diseases in growth hormone receptor/binding protein knockout mice.  These changes of fatal neoplasms are similar to the effects observed with calorie restriction and therefore could possibly be a major contributing factor to the extended life span observed in the GHR/BP KO mice. [114] Overall, 2009 was an exciting year for increasing our understanding of aging and its relationship to age-related disease, and developing promising strategies and candidates for pharmacological interventions into the aging process. Several approaches in combination with drugs and diet may slow aging, although not making it negligible [115].

## Acknowledgments and announcements

We apologize to the authors whose important publications were not discussed due to space limitations or were simply overlooked. Here we referenced only papers published in the 2009 calendar year. That was not an easy task given that most of publications are the continuation of or based on previous research. We expect to make this a tradition to publish ‘the year overview' every year. The next year, the task will be easier, given that "Aging in 2010" will be the continuation of "Aging in 2009".

Please e-mail your reprints to us in December 2010 (editors@impactaging.com) or, even better, please submit your best manuscripts for publication in ***Aging*** at papers@impactaging.com.
